# Melanisation of *Aspergillus terreus*—Is Butyrolactone I Involved in the Regulation of Both DOPA and DHN Types of Pigments in Submerged Culture?

**DOI:** 10.3390/microorganisms5020022

**Published:** 2017-05-04

**Authors:** Elina K. Palonen, Sheetal Raina, Annika Brandt, Jussi Meriluoto, Tajalli Keshavarz, Juhani T. Soini

**Affiliations:** 1 Biochemistry, Faculty of Science and Engineering, Åbo Akademi University, Artillerigatan 6, Åbo FI-20520, Finland; annika.brandt@abo.fi (A.B.); jussi.meriluoto@abo.fi (J.M.); 2 Department of Life Sciences, University of Westminster, London W1W 6UW, UK; rainas2@googlemail.com (S.R.); t.keshavarz@westminster.ac.uk (T.K.); 3 Faculty of Life Sciences and Business, Turku University of Applied Sciences, Lemminkäinengatan 30, Åbo FI-20520, Finland; juhani.soini@turkuamk.fi

**Keywords:** *Aspergillus terreus*, filamentous fungi, pigment, melanin, butyrolactone I, transcriptome sequencing, gene expression, NR-PKS cluster

## Abstract

Pigments and melanins of fungal spores have been investigated for decades, revealing important roles in the survival of the fungus in hostile environments. The key genes and the encoded enzymes for pigment and melanin biosynthesis have recently been found in Ascomycota, including *Aspergillus* spp. In *Aspergillus terreus*, the pigmentation has remained mysterious with only one class of melanin biogenesis being found. In this study, we examined an intriguing, partially annotated gene cluster of *A. terreus* strain NIH2624, utilizing previously sequenced transcriptome and improved gene expression data of strain MUCL 38669, under the influence of a suggested quorum sensing inducing metabolite, butyrolactone I. The core polyketide synthase (PKS) gene of the cluster was predicted to be significantly longer on the basis of the obtained transcriptional data, and the surrounding cluster was positively regulated by butyrolactone I at the late growth phase of submerged culture, presumably during sporulation. Phylogenetic analysis of the extended PKS revealed remarkable similarity with a group of known pigments of *Fusarium* spp., indicating a similar function for this PKS. We present a hypothesis of this PKS cluster to biosynthesise a 1,8-dihydroxynaphthalene (DHN)-type of pigment during sporulation with the influence of butyrolactone I under submerged culture.

## 1. Introduction

The filamentous fungi of *Aspergillus* species are known to cause several diseases and to contain pathogenic features with objects ranging from plants to humans. These fungal features include resistance to environmental damage sources such as UV, heat, detergents, phagocytosis and antimicrobial drugs. One of the factors involved in these antagonistic properties is fungal sporulation and the corresponding resistance improving characteristics of the spores, including pigments and melanins [1,2,3,4,5,6]. The biosynthesis pathways of fungal pigment and melanin polymers have been divided into two classes, DHN (1,8-dihydroxynaphthalene) or DOPA (3,4-dihydroxyphenylalanine) pathways. The classification of these types is, however, diverged into two methods, either based on the identification of the pathway intermediates, or on the observed effects of applying specific intermediate enzyme inhibitors. Specifically, the DHN classification is either based on the identification of naphthopyrone precursors or on the effect of inhibitors—tricyclazole or phthalide—targeted to hydroxynaphthalene reductase with classical short-chain dehydrogenase/reductase (SDR) and Rossmann fold domains. The biogenesis of this type of melanin usually begins with polyketide synthesis, followed by tailoring steps and polymerisation. The classification of the other melanin type, DOPA, is based on the presence of tyrosine or 3,4-dihydroxyphenylalanine precursors, or on the involvement of tyrosinase enzyme, indicated with inhibitors kojic acid and tropolone. The resulting melanin intermediates of this DOPA pathway are commonly polymerised as well [6,7,8,9,10]. So far, the biosynthesis and polymerisation pathways as well as the specific morphological locations of diverse pigments are still unrevealed concerning many filamentous fungi. However, both DHN-type of conidial pigments and DOPA-type of melanins have been discovered in few *Aspergillus* species, although the DHN-type of pigments are presumed to be more common [6,11,12,13,14].

The key enzymes in producing pigments and melanins in *Aspergillus terreus* have been searched for in several studies with no resulting identifications regarding polyketide synthases (PKS) [14,15,16], as far as we know. In contrast, a non-ribosomal peptide synthetase (NRPS)-like enzyme MelA was revealed to synthesise an uncommon aspulvinone E-derived Asp-melanin together with a tyrosinase TyrP [17,18], indicating the production to be of DOPA-type. Nevertheless, the pigmentation pathways of *A. terreus* are still mysterious, being only partially discovered. Pal et al. demonstrated the presence of a DHN-type of pigment using specific inhibitors, both on static culture as well as in submerged growth conditions [14]. Intriguingly, Schimmel et al. reported increased sporulation along with secondary metabolism of *A. terreus* in submerged culture conditions, as a result of supplementing butyrolactone I, which was later implicated as a quorum sensing inducing molecule in *A. terreus* [19,20,21]. Butyrolactone I was recently suggested to be involved in the gene expression control of the key regulators of conidiation (*brlA*, *abaA* and *wetA*) as well as the global regulator *laeA* [22] in accordance with the previous study of Schimmel et al. [19]. However, pigmentation has not been examined in the same growth conditions, to our knowledge.

In this study, we describe a potential, non-reducing polyketide synthase (NR-PKS) with a non-canonical domain structure within Aspergilli, and the surrounding gene cluster, which were revealed through an in-depth analysis of the recently sequenced transcriptome of *A. terreus* strain MUCL 38669 [22]. The gene cluster transcripts appeared to be only partially annotated in the *A. terreus* strain NIH2624, leading to a further examination of the transcripts. We present a hypothesis of the function for this gene cluster to be involved in a DHN-type pigmentation pathway, in advance of the necessary further investigations. We display the transcriptional levels and gene expression profiles of both the known DOPA-like as well as the suggested DHN-like pigment clusters during the developmental growth phases of the submerged *A. terreus* culture. We suggest that butyrolactone I, as an indicated quorum sensing molecule, plays a regulative role in the pigmentation processes based on large-scale gene expression and whole transcriptome sequencing results.

## 2. Materials and Methods

### 2.1. Strain, Chemicals and Culture Conditions

All culture materials, including the *Aspergillus terreus* strain MUCL 38669, are the same as was used previously [20,21,22]. *A. terreus* MUCL 38669 was cultured under shaken, submerged growth conditions in three biological replicates for nine days. The growth conditions were the same as in previous studies of secondary metabolism [20,21]. Shortly, *A. terreus* MUCL 38669 spores were maintained on yeast and malt extract (YME) agar slants. Collected spores (final concentration $10^{7}$/mL) had been incubated in 100 mL of inoculation medium for 25 h at 27 ℃. In addition, 100 mL of glucose, peptonised milk, yeast extract and lactose containing (GPY-L) production medium (pH 7.4) was inoculated with 10 mL of the inoculation medium and incubated at 27 ℃ for 216 h.

#### Addition of Butyrolactone I

Exogenous butyrolactone I was added at 24 h, 96 h and 120 h post inoculation (test sets 1, 2 and 3, respectively) and each of the test sets as well as the control set (no butyrolactone I added) were sampled at 24 h, 48 h, 96 h, 120 h, 144 h and 216 h post inoculation. The exogenous butyrolactone I was dissolved in ethanol and added to the test cultures to a final concentration of 100 nM.

### 2.2. Gene Expression Analysis Using Microarrays

The total RNA was derived from a previous study [20] where *A. terreus* mycelia was sampled at the 6 time points and was stored at −80 ℃. The microarray gene expression data was obtained and analysed as described in our related study [22]. The analysis contained one unusual step regarding the 60-mer oligonucleotide microarray probes that had been designed based on the available genomic sequence of strain NIH2624 [23]. Additional bioinformatic steps were applied to exclude the unreliable probes by alignment based filtering using BLASTN software (version 2.2.29+, National Centre for Biotechnology Information (NCBI), Bethesda, MD, USA) [24,25] prior to the further deep analysis, due to the observed nucleotide-level differences between the *A. terreus* strains NIH2624 and MUCL 38669 (details in our related study [22]). The microarray experiment was performed in three biological replicates and in four technical replicates on probe level. Differential gene expression (∣log${}_{2}$FC∣ > 0.5) was considered as statistically significant if adjusted *p* < 0.05.

### 2.3. Strand-Specific Transcriptome Sequencing and Analysis

The assembled transcriptome sequence data was obtained from our related study [22]. Shortly, the method used for the synthesis of strand-specific double stranded cDNA (ds cDNA) from the total RNA was a modified combination of protocols presented by Marioni et al. [26], Parkhomchuk et al. [27], Levin et al. [28] and the standard Illumina mRNA preparation protocol (Preparing Samples for Sequencing of mRNA, Part No. 1004898 Rev. A, Illumina Inc., San Diego, CA, USA) due to the lack of strand-specificity in the contemporary standard mRNA sequencing protocols. The total RNA to be sequenced was extracted from the *A. terreus* mycelia samples taken at six time points of the culture where butyrolactone I had been added at 120 h p.i., obtained from the submerged culture of a previous study [20]. This set of total RNA was pooled to acquire the required amount of strand-specific ds cDNA for the high throughput DNA sequencing. The detailed description of the RNA preparation and the sequence assembly is explained in our related study [22].

The transcriptome analysis was performed as in our related study [22] and composed of the following steps, in short. The obtained transcripts along with the obtained sequence reads of *A. terreus* MUCL 38669 were aligned with the strain NIH2624 annotated genome and viewed using Integrative Genomics Viewer (IGV) (version IGV_2.3.23, Broad Institute, Cambridge, MA, USA) [29,30,31]. The resulting mapped reads were quantified and normalised using Cufflinks tools Cuffquant and Cuffnorm (version 2.2.1, University of Washington, Seattle, WA, USA) [32], giving the number of fragments per kilobase of exon per million reads mapped (FPKM), representing the accumulation of the transcripts of the pooled RNA samples (pooled FPKM).

### 2.4. Data Availability

The resulting complete transcript sequences analysed in this study were deposited at the National Centre for Biotechnology Information (NCBI) GeneBank database under the following accession numbers: KX470747 (*pgmB*), KX470748 (*pgmD*), KX470749 (*pgmE*), KX470750 (*pgmF*), KX470751 (*pgmG*), KX470752 (*pgmH*), and KX470753 (*melA*). The putative PKS sequence, *pgmA*, is deposited as a third-party annotation with the accession number BK009975. The raw transcriptome sequence data and microarray gene expression raw data of *A. terreus* MUCL 38669 have been submitted to the Sequence Read Archive (SRA) and Gene Expression Omnibus (GEO) databases of NCBI. The obtained accession numbers are PRJNA360953 (BioProject) and GSE93552 (GEO database).

## 3. Results

### 3.1. A Non-Canonical PKS Gene Cluster in A. terreus

**Figure 1:**
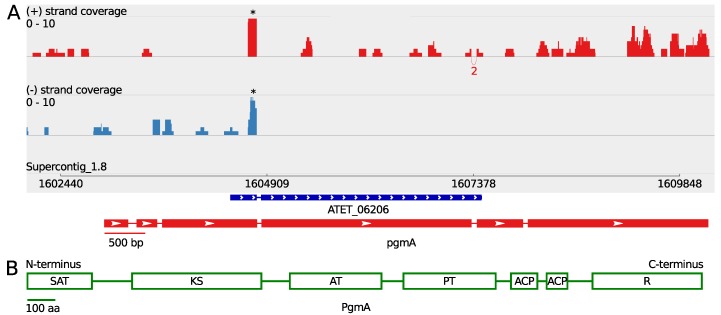
The predicted non-reducing polyketide synthase PgmA. (**A**) sashimi plot of the *pgmA* gene describing the strand-specific alignment coverage of the read sequences of *A. terreus* MUCL 38669 over the corresponding genomic region of NIH2624 supercontig_1.8, which was obtained using the Integrative Genomics Viewer (IGV) software [29,30,31]. The curved splice junctions represent the number of spliced reads indicating an intron at that specific location. The predicted gene structure is shown below the sashimi plot in the same scale as the plot; (**B**) the predicted protein domains of the translated sequence of PgmA: Starter unit:ACP transacylase (SAT), Beta-ketoacyl synthase (KS), Acyl transferase (AT), Polyketide product template (PT), Acyl carrier (ACP), Thioester reductase (R). * These coverage peaks are due to an erroneously aligned short part of a longer transcript located to a gene ATEG_06102.

Exogenous supplementation of butyrolactone I has been suggested to increase sporulation under submerged growth conditions [19] and was recently further investigated on a transcriptomic level in our related study [22]. While analysing the sequenced transcriptome in order to illuminate the predicted secondary metabolite clusters, an intriguing cluster was encountered, containing an only partially annotated core PKS gene (ATEG_06206 in strain NIH2624, subsequently called *pgmA*). The transcriptome sequence data indicated a significantly longer transcript for this PKS gene. A computational GENSCAN [33] analysis of the surrounding genomic region of the PKS gene revealed the length of this open reading frame (ORF) to be 7232 bp in accordance with the indicated length of the partially sequenced transcript (Figure 1 and Table 1). Translation using ExPASy Web Server [34] and the subsequent InterPRO protein domain prediction [35] revealed the protein to contain several domains that classify this PKS protein as a non-reducing polyketide synthase. The domain structure prediction revealed following domains: starter unit:ACP transacylase domain (SAT) on N-terminus, beta-ketoacyl synthase domain (KS), acyl transferase domain (AT), polyketide product template domain (PT), two successive acyl carrier domains (ACP) and thioester reductase domain (R) in the C-terminus, suggesting an exquisite NR-PKS subclass amongst Aspergilli (Figure 1).

**Figure 2:**
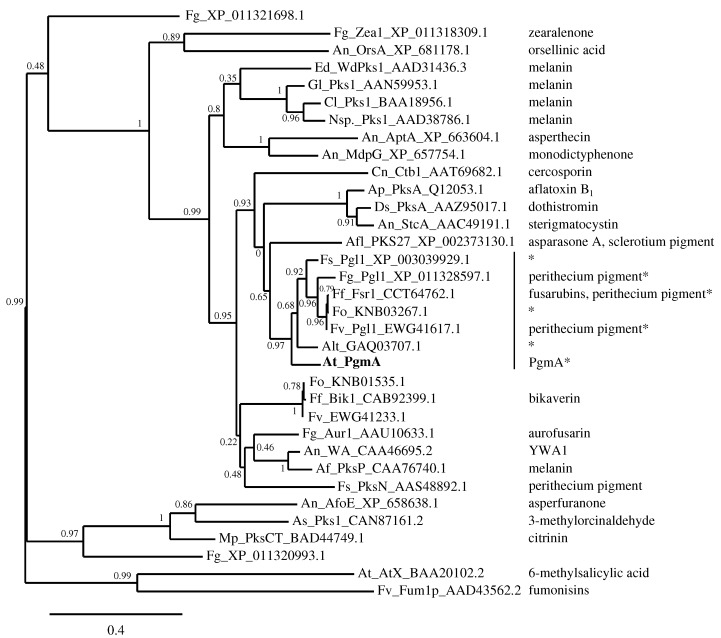
Phylogeny of different fungal polyketide synthases including pigment synthases. The range of polyketide synthases chosen for this analysis is based on a study of Studt et al. [37]. The extracted ketoacyl synthase domains of the polyketide synthases were compared and aligned to obtain a phylogram as described in our related study [22]. The chemical product of the corresponding polyketide synthase (PKS) is given on the right side if characterised, to our knowledge. The scale bar represents a 0.4 residue change per site. No outgroup was used. The organisms used in the phylogram: Af: *A. fumigatus*, Afl: *A. flavus*, Alt: *A. lentulus*, An: *A. nidulans*, Ap: *A. parasiticus*, As: *Acremonium strictum*, At: *A. terreus*, Cl: *Colletotrichum lagenaria*, Cn: *Cercospora nicotianae*, Ds: *Dothistroma septosporum*, Ed: *Exophiala dermatitidis*, Ff: *Fusarium fujikuroi*, Fg: *F. graminearum*, Fo: *F. oxysporum*, Fs: *F. solani*, Fv: *F. verticillioides*, Gl: *Glarea lozoyensis*, Mp: *Monascus purpureus*, Nsp.: *Nodulisporium* sp. strain ATCC74245. The protein sequences (except for the PgmA) were obtained from the following databases: National Centre for Biotechnology Information (NCBI) RefSeq, GenBank, DNA Data Bank of Japan (DDBJ), European Molecular Biology Laboratory (EMBL) and UniProtKB. * PKS domain structure is: SAT-KS-AT-PT-ACP-ACP-R.

A BLASTP search [36] displayed good similarity with a group of NR-PKS proteins of *Fusarium* species as the best match along with *Aspergillus lentulus* and *Aspergillus glaucus*, having residue identities between 58% and 63% and full-length alignment. A closer look at these BLASTP results revealed all of the proteins in this group to have the same domain structure with the PgmA protein whereas only *A. lentulus* and *A. glaucus* amongst *Aspergillus* species has a PKS protein with a similar domain structure to our knowledge. Four of the proteins of the group of *Fusarium* NR-PKS proteins with the same domain structure are known to produce perithecium pigments [37,38]. Specifically, these proteins are *F. graminearum* Pgl1 (XP_011328597.1), *F. fujikuroi* fusarubin producing Fsr1 (CCT64762.1), *F. verticillioides* Pgl1 (EWG41617.1) and *F. solani* Pgl1 (XP_003039929.1). A phylogenetic study of KS domains of several Ascomycete NR-PKS proteins, PgmA included, revealed homology to these core PKS enzymes of perithecium pigment biosynthesis (Figure 2), predicting a similar function to *A. terreus* PgmA protein. A closer look on the surrounding genes of *pgmA* revealed a cluster of nine genes in total with predicted domains that are common for enzymes in secondary metabolites producing clusters (Table 1 and Table 2). Each of the cluster genes has a complete or partial transcript coverage, indicating co-expression of these genes and some activity for this cluster in these growth conditions while the genes up- and downstream of this cluster displayed no coverage, confirming the cluster form (Figures S1–S3).

A further BLASTP analysis also revealed similarity with the fusarubin biosynthesis cluster members of *F. fujikuroi*, having some genes in common with this cluster of *A. terreus*, in addition to the predicted core biosynthesis PKS gene. Specifically, the clusters have a putative O-methyltransferase ATEG_06203 (subsequently called *pgmB*), a putative quinone reductase ATEG_06209 (subsequently called *pgmF*) and a transcription factor ATEG_06205 (subsequently called *pgmR*) with similarity on the amino acid level and additionally a putative short-chain dehydrogenase/reductase ATEG_06207 (subsequently called *pgmD*) with a shared domain structure (Table 2). The central, multi-functional tailoring enzyme of the fusarubin cluster, Fsr3 with a flavin adenine dinucleotide (FAD) binding monooxygenase domain, has no clearly orthologous enzyme in this *A. terreus* cluster, whereas the cluster includes a putative cytochrome p450 monooxygenase encoding gene ATEG_06204 (subsequently called *pgmC*), possibly having a similar function in the *A. terreus* biosynthesis pathway (Table 2). The *A. terreus* cluster appears to include three additional genes when compared to fusarubin biosynthesis gene cluster, namely, a putative S-adenosyl-L-methionine (SAM)-dependent methyltransferase ATEG_06208 (subsequently called *pgmE*), a putative major facilitator superfamily (MFS) permease ATEG_06210 (subsequently called *pgmG*) and a putative FAD-binding CO dehydrogenase ATEG_06211 (subsequently called *pgmH*) (Table 2). Taken together, we report a transcriptionally updated and computationally confirmed gene cluster annotation containing a non-canonical NR-PKS *pgmA*, being similar to pigment biosynthases amongst *Fusarium* species on protein level. All of these cluster genes being co-expressed suggests a secondary metabolite, possibly a pigment, to be biosynthesised in these submerged growth conditions under enhanced butyrolactone I influence.

### 3.2. Butyrolactone I Addition Reveals Opposite Gene Expression Profiles for the Pgm Cluster Genes and DOPA-Type Melanin Biosynthesis Genes

**Figure 3:**
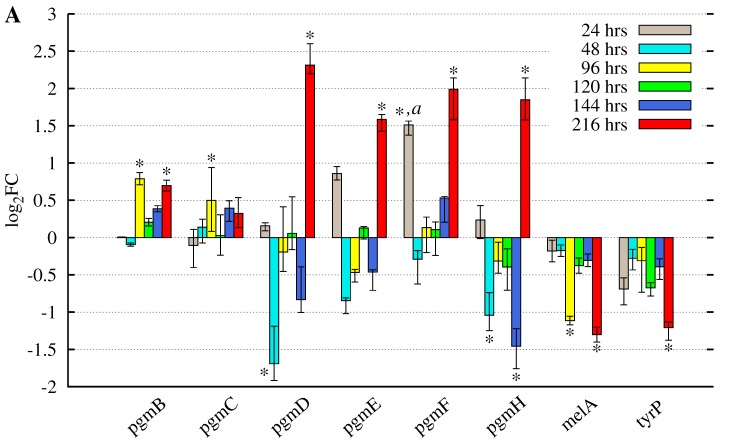

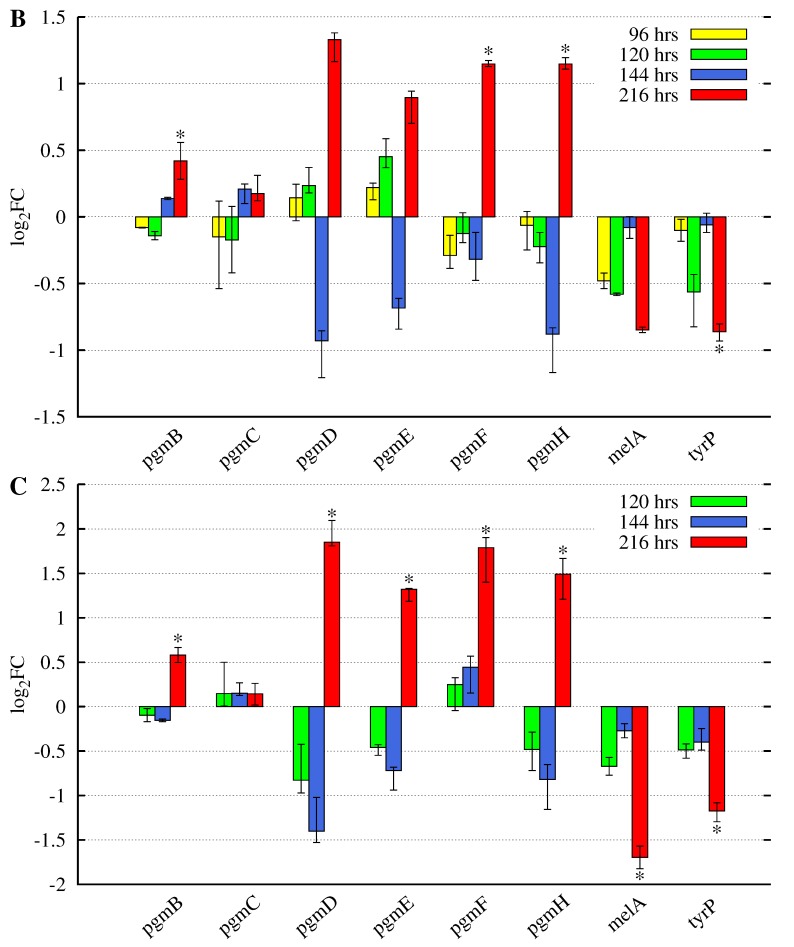
Gene expression analysis of the *A. terreus pgm* PKS cluster and *asp-melanin* genes under influence of butyrolactone I. Exogenous butyrolactone I was added to the submerged culture of *A. terreus* MUCL 38669 at (**A**) 24 h; (**B**) 96 h; and (**C**) 120 h p.i. to the final concentration of 100 nM. The lack of some cluster genes is due to the nucleotide level differences between strains MUCL 38669 and NIH2624 or the low transcript coverage obtained. The bars represent the median values of the log${}_{2}$FC of treated samples (butyrolactone I was added) versus control samples (no butyrolactone I added) and the error bars represent the maxima and minima of these log${}_{2}$FC values. * At least one of the technical replicates indicates statistically significant up- or downregulation of the three biological replicates (adjusted *p*-value ≤0.05 and ∣log${}_{2}$FC∣≥0.5). ${}^{a}$ The apparently statistically significant upregulation at 24 h post inoculation may not be biologically significant due to the low culture density at the start of exponential growth phase.

Recently, an uncommon melanin biosynthesis pathway of DOPA-type in *A. terreus* has been revealed to be composed of an NRPS-like biosynthase MelA (ATEG_03563) and a tyrosinase TyrP (ATEG_03564) [17,18]. In the sequenced transcriptome data, both of these genes were also expressed (Figure S4), with *tyrP* transcripts having lower pooled accumulation level in comparison to *melA* and the majority of genes encoding the tailoring enzymes of the *pgm* cluster (Table 1). The observation that both of these gene clusters were expressed in the growth conditions used in this study led to a closer investigation of the previously obtained and accuracy improved in-depth microarray gene expression results. The microarray accuracy was improved on a probe sequence level, which was evaluated with the transcriptome sequence data as conducted in our related study [22]. Reliable gene expression data was available for six of the nine *pgm* cluster genes and both of the two DOPA-type melanin biosynthesis encoding genes (Figure 3). The addition of butyrolactone I, regardless of the time point of addition, resulted in opposite gene expression patterns of the *asp-melanin* and *pgm* cluster genes. The DOPA-type *asp-melanin* biosynthesis genes were increasingly downregulated towards the late growth phase: at 216 h post inoculation, it was statistically significant, whereas the *pgm* cluster genes were statistically significantly upregulated at the end of late growth phase. In particular, the *pgmD*, putative short-chain dehydrogenase/reductase, was significantly upregulated with a log${}_{2}$FC value above 2 when butyrolactone I was added at 24 h post inoculation. Some of these *pgm* cluster genes were also statistically significantly downregulated prior to the end of late growth phase, i.e., before 216 h p.i. (Figure 3).

Taken together, the observed gene expression patterns indicate both direct and non-direct regulative roles for butyrolactone I concerning both of these two clusters, in these submerged growth conditions. The supplementation during the exponential growth phase led to both direct and non-direct regulation (Figure 3A), whereas the addition appears to have effect in a non-direct manner with an interval of 120 h when butyrolactone I was added during the middle growth phase (at 96 h p.i.), while the addition during the late phase (at 120 h p.i.) resulted in an interval of 96 h before the displayed positive effect at 216 h p.i. (Figure 3B,C). The melanin producing genes display a similar intervalled expression pattern with one remarkable exception: the regulation conducted by butyrolactone I appears as continuously negative. Furthermore, the effect of the added butyrolactone I was most significant at the late growth phase regardless of the time point of addition unifying the presumably opposite gene expression regulation between both gene clusters.

## 4. Discussion

### 4.1. The Similarity of A. terreus Pgm Cluster with Pigment Biosynthesis Genes of Aspergilli and F. fujikuroi

Fungal melanins are often complex polymers and divided into two different types (DHN and DOPA), depending on the characteristics of the biosynthesis pathways, being either produced intermediates or involved enzymes. Regarding *Aspergillus* species, the DHN-type of pigments are presumed to be more common, but DOPA-type of melanins have also been observed. *A. fumigatus* contains a DHN-type of conidial pigment biosynthesis cluster including an NR-PKS Alb1/PksP (with domains SAT-KS-AT-PT-ACP-ACP-TE/CLC) as well as Arp2, a hydroxynaphthalene reductase, common in this type of pigment cluster [41]. In *A. nidulans*, the homologous NR-PKS WA—with the same domains as *A. fumigatus* Alb1/PksP—has been shown to be involved in conidial pigmentation but is not genomically located in any specific cluster [11]. Both of these PKS enzymes accept malonyl-CoA as a precursor, whereas *A. fumigatus* cluster produces a pentaketide 1,3,6,8-tetrahydroxynaphthalene (T4HN; naphthalene-1,3,6,8-tetrol) to be polymerised into melanin, while *A. nidulans* produces YWA1 (heptaketide naphthopyrone)—another form of the initial precursor of the melanisation pathway of *A. fumigatus* [42,43]. *A. flavus* contains three separate PKS homologues of *A. fumigatus* Alb1/PksP, the most similar one (PksP) being in a similar gene cluster while the other two homologues are located in diverse gene clusters. One of them was shown to be the key polyketide synthase—domains SAT-KS-AT-PT-ACP-TE/CLC, having acetyl-CoA and malonyl-CoA as precursors—that produces putative anthraquinone intermediates as a presumed source for a sclerotia-specific pigment. The suggested pigment source was either a dehydrated form of anthraquinone asparasone A (1,3,6,8-tetrahydroxy-2-(1’-hydroxy-3’-oxobutyl)-anthraquinone) or a reduced form of anthraquinone [12]. There are also few DOPA-types of melanins reported to be biosynthesised, specifically in *A. nidulans* and *A. flavus*—with the intermediates and precursors remaining unknown [13,14], whereas *A. fumigatus* has been observed to produce pyomelanin through a tyrosine degradation pathway, having benzoquinoneacetate as the precursor for polymerisation [44]. *A. terreus* has been reported to biosynthesise aspulvinone E-derived melanin by an NRPS-like enzyme (MelA) with 4-hydroxyphenylpyruvate as a precursor and subsequent hydroxylation of aspulvinone E ((5Z)-4-hydroxy-3-(4-hydroxyphenyl)-5-[(4-hydroxyphenyl)methylidene]furan-2-one) by tyrosinase (TyrP) prior to polymerisation [17,18], classifying the Asp-melanin as DOPA-type.

**Figure 4:**
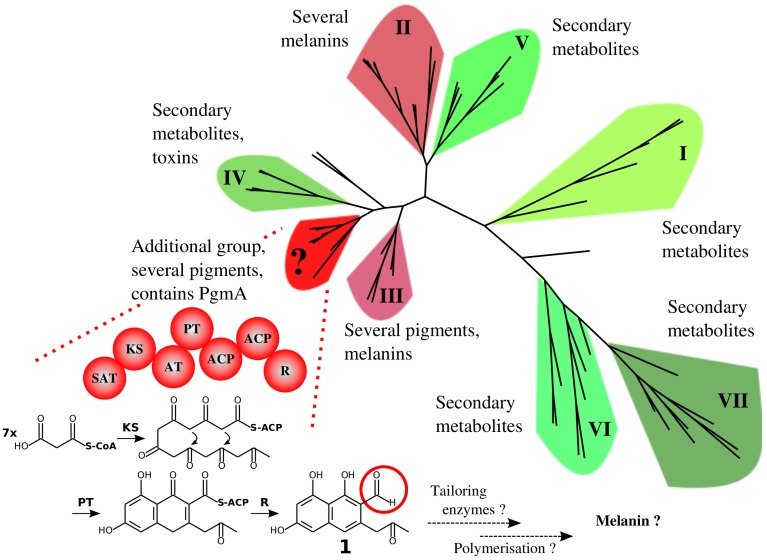
A phylogenetic scheme of various non-reducing polyketide synthases amongst *Ascomycota* phylum, dividing into eight groups based on the similarity of the ketosynthase domain sequences. The suggested hypothetical action mechanism for the majority of the NR-PKS enzymes amongst the additional group is as proposed by Awakawa et al., resulting in the intermediate 1; 3-acetonyl-1,6,8-trihydroxy-2-naphthaldehyde [38]. See a more detailed Figure S5 for the included NR-PKS proteins. The group classification of I-VII is based on the classes presented in the study of Ahuja et al., and the range of NR-PKS enzymes included in this analysis is based on the studies of Studt et al. and Ahuja et al. [37,45]. The extracted ketoacyl synthase domains of the polyketide synthases were compared and aligned to obtain the phylogram as described in our related study [22]. No outgroup was used. The protein sequences were obtained from the following databases: NCBI RefSeq, GenBank, DDBJ, EMBL and UniProtKB.

Amongst Ascomycota, the NR-PKS enzymes have been divided into seven different groups, two of which contain enzymes involved in the biosynthesis of melanins and pigments [45]. When these groups are phylogenetically analysed together with the NR-PKS enzymes included in the phylogram presented in the study of Studt et al. [37] (Figure 2), an additional group within NR-PKS enzymes is formed containing several enzymes involved in the biosynthesis of pigments and melanins of *Fusarium* species, fusarubin included, and the asparasone A-derived pigment of *A. flavus*. Furthermore, the predicted PgmA of *A. terreus* appears to be part of the additional group (Figure 4 and Figure S5). The majority of the members of this additional group share the predicted domain structure—SAT-KS-AT-ACP-ACP with the thioester reductase (R) domain on the C-terminus—resulting in a terminal aldehyde on the intermediate (heptaketide naphthaldehyde; 3-acetonyl-1,6,8-trihydroxy-2-naphthaldehyde (1)) of the fusarubin biosynthesis pathway as proposed by Awakawa et al. [38] and Studt et al. (Figure 4) [37]. Thus far, this reductive release mechanism appears to be rather uncommon amongst the non-reducing PKS enzymes of *Aspergillus* species—especially regarding the known pigment biosynthesis pathways. The *A. terreus pgm* cluster with the presumed core NR-PKS gene as described in this study contains also a putative short-chain dehydrogenase/reductase gene (*pgmD*) with predicted classical SDR family and NADB_Rossmann superfamily protein domains (Table 2), displaying similarity with the domains of *A. fumigatus* Arp2 of the pigment cluster that contains the core PKS Alb1 [41]. The domain similarity of encoded PgmD with this common hydroxynaphthalene reductase as well as the same domain structure of the predicted PgmA in comparison to the *F. fujikuroi* Fsr1, the core NR-PKS enzyme of the fusarubin pigment cluster [37], sparks a hypothesis of this *A. terreus pgm* cluster to produce a DHN-type of pigment.

### 4.2. Hypothesised A. terreus DHN-Type of Pigment and the Known DOPA-Type of Melanin May Be Biosynthesised under Different Growth Conditions

In contrast to the observed DOPA-type melanin in *A. terreus* [17,18], Pal et al. [14] has reported melanin production to occur through the DHN pathway. This was demonstrated by adding the hydroxynaphthalene reductase inhibitors, tricyclazole and phthalide, which resulted in albino culture during static growth conditions, while the actual gene cluster still remained unknown. Melanin was also detected in the filtrate of submerged culture while no melanin was detected in the culture biomass. In addition, no production of DOPA-type of melanin was detected by using inhibitors kojic acid and tropolone [14]. In contradiction, the effects of deletion of a DOPA pathway gene (*melA*) [17,18] and the addition of the DHN inhibitors [14] were both observed on static culture conditions to result in albino culture in both cases, which implies two separate melanin pigment biosynthesis pathways for *A. terreus*. This is due to the fact that no effect was observed when DOPA inhibitors were added in the same growth conditions as DHN inhibitors were added [14]. In particular, the observation that DOPA inhibitors had no effect during static growth conditions by Pal et al. [14], while the observations by Guo et al. and Geib et al. [17,18] indicate the presence of DOPA pathway on static culture conditions, emphasises the importance of the growth conditions including production media used in melanin production. In this study, the used culture conditions being shaken and submerged, with exogenous addition and enhanced production of butyrolactone I (demonstrated in [20,21]), result in both *pgm* and *asp-melanin* clusters being expressed on a transcript level with opposing gene expression patterns (Figure 3, Figures S1, S2 and S4). These controversial gene expression profiles support the hypothesis of biogenesis of a DHN-type pigment by the *pgm* cluster.

### 4.3. The Suggested DHN-Like Pigment of A. terreus May Be Related to Conidiophores as Well

In our related study [22], the gene expression patterns of the conidiation regulating genes, *abaA* and *wetA*, are described to be negatively regulated prior to the end of last growth phase, while *abaA* is positively regulated at the last growth phase, 216 h p.i., in the same growth conditions as used in this study. The gene expression profiles of the presumed pigment cluster *pgm* (Figure 3) are quite similar with especially the gene expression pattern of the phialide emergence inducing gene, *abaA*, whereas the gene expression profiles of the DOPA-type Asp-melanin biosynthesis genes have contradictory forms. This indicates the predicted DHN-like pigment to be biosynthesised during the last steps of conidiation, phialide development followed by conidia maturation, while the Asp-melanin is presumably produced in a lesser amount. The gene expression profile and the convenient chronological order led us to further hypothesise regarding the morphological location of the suggested DHN-like melanin to be in the conidiophores in these submerged and shaken conditions under enhanced butyrolactone I biogenesis. This is in accordance with an observation of increasingly brown submerged culture towards the late phase, i.e., the ninth day of growth (unpublished observation). However, further studies are required to confirm this.

The question whether this *pgm* gene cluster produces a DHN-like pigment, instead of some unrevealed secondary metabolite, may be explained by the gene expression profile of the secondary metabolism inducing LaeA, which was displayed in our related study [22]. The obtained *laeA* gene expression profile appears to be chronologically opposed to the revealed expression pattern of *pgm* cluster (Figure 3). In our related study, *laeA* displayed significant upregulation by butyrolactone I prior to the late growth phase (at 48, 120 and 144 h p.i.), while it was unaffected at the late growth phase (216 h p.i.), indicating the increased secondary metabolism to occur prior to enhancing the gene expression of the *pgm* cluster. The biogenesis of the secondary metabolite lovastatin has been observed to be increased by butyrolactone I during the middle growth phase in the same growth conditions as used in this study [19,20], in agreement with the upregulated *laeA* gene expression profile [22]. Considering these observed occurrence patterns in chronological order, the activity of the *pgm* cluster appears to be enhanced after the increase in secondary metabolism in these submerged culture conditions under the influence of increased butyrolactone I biogenesis.

## 5. Conclusions

In conclusion, we provide a hypothesis suggesting the new, non-canonical NR-PKS PgmA amongst Aspergilli, being an ortholog of *F. fujikuroi* fusarubin core synthase, Fsr1, to be a key pigment biosynthase with the reductase domain on the C-terminus. In addition, we suggest the cluster in the vicinity of this core gene, *pgmA*, to contain a hydroxynaphthalene reductase-like short-chain dehydrogenase/reductase PgmD, along with five co-expressed tailoring enzymes. Integrated with our related study of *A. terreus* conidiation during submerged culture, the results of this study indicate increased biogenesis of a conidia related DHN-type of pigment under submerged growth conditions with increased butyrolactone I production. To confirm the obtained results and this hypothesised conidial pigment production, further comprehensive studies including quantitative RNA-sequencing and molecular as well as morphological analyses are necessary.

## Figures and Tables

**Table 1:** Information on the *A. terreus pgm* gene cluster and the DOPA type melanin genes.

Gene	Gene ${}^{a}$ Length (bp)	ORF Length (bp)	Pooled FPKM	Coverage Max. ${}^{b}$ Sense Strand
*pgmB*	1448	1329	3.9	23 ${}^{c}$
*pgmC*	1443 ${}^{d}$	1443 ${}^{d}$	1.1	6
*pgmR*	1284 ^e^	1284 ^e^	0.13	2
*pgmA*	7232 ^e^	6897 ^e^	0.65	8
*pgmD*	1003	939	4.7	44 ${}^{c}$
*pgmE*	1008	1008	15	91 ${}^{c}$
*pgmF*	1071	1071	290	1634 ${}^{c}$
*pgmG*	1967	1683	56	333 ${}^{c}$
*pgmH*	1714	1479	12	81 ${}^{c}$
*melA*	2779	2779	6.0	38 ${}^{c}$
*tyrP*	1230 ${}^{d}$	1071 ${}^{d}$	1.3	14

${}^{a}$ based on the sequenced ORF’s 5${}^{\prime }$ and 3${}^{\prime }$ locations on the *A. terreus* NIH2624 genome assembly; ${}^{b}$ represents the number of overlapping reads’ nucleotides at one nucleotide site of the transcript obtained from the pooled RNA samples; ${}^{c}$ complete read coverage; ${}^{d}$ confirmed with GENSCAN Web Server [33]; ^e^ predicted with GENSCAN Web Server [33]; DOPA: 3,4-dihydroxyphenylalanine; ORF: open reading frame; FPKM: fragments per kilobase of exon per million reads mapped.

**Table 2:** Amino acid identity and InterPRO domains of the *A. terreus* Pgm cluster and *F. fujikuroi* fusarubin cluster ${}^{a}$.

Gene	Predicted Molecular Function	InterPRO Annotation	Domains	ID %	Fusarubin Gene
PgmB	O-methyltransferase	IPR016461 ${}^{d}$	O-methyltransferase, COMT-type	41	Fsr2 ${}^{f}$
		IPR011991 ${}^{d}$			
		IPR029063 ${}^{d}$			
		IPR001077 ${}^{d}$			
PgmC ${}^{b}$	Cytochrome p450 monooxygenase	IPR001128	Cytochrome P450 E-class, group 1	NA ^e^	Fsr3 ${}^{g}$
		IPR002401			
		IPR017972			
PgmR ${}^{b}$	Aflatoxin biosynthesis regulatory protein-like	IPR002409	Aflatoxin biosynthesis regulatory protein	31	Fsr6 ${}^{h}$
		IPR001138 ^d^			
PgmA ${}^{b,c}$	nonreducing polyketide synthase; NR-PKS	IPR032088 ${}^{d}$ IPR020841 ^d^ IPR020801 ^d^ IPR030918 ^d^ IPR009081 ^d^ IPR013120 ^d^	Starter unit:ACP transacylase Beta-ketoacyl synthase Acyl transferase Polyketide product template Acyl carrier protein-like Thioester reductase-like	59	Fsr1 ${}^{i}$
PgmD	short-chain	IPR002347 ${}^{d}$	Short-chain	NA ^e^	Fsr5 ${}^{j}$
	dehydrogenase/reductase	IPR016040 ${}^{d}$	dehydrogenase/reductase		
PgmE	SAM-dependent	IPR029063	SAM-dependent	NA	NA
	methyltransferase		methyltransferase		
PgmF	quinone reductase	IPR002085 ${}^{d}$ IPR011032 ${}^{d}$ IPR020843 ${}^{d}$ IPR013154 ${}^{d}$ IPR016040 ${}^{d}$	Alcohol dehydrogenase GroES-like Enoylreductase domain Alcohol dehydrogenase	26	Fsr4 ${}^{k}$
PgmG	MFS family permease	IPR011701	Major facilitator superfamily	NA ^e^	NA ^e^
		IPR020846			
PgmH	FAD/FMN-binding CO dehydrogenase	IPR016167	FAD-binding, type 2	NA ^e^	NA ^e^
		IPR016166	FAD linked oxidase		
		IPR006094	CO dehydrogenase flavoprotein-		
		IPR016169	like, FAD-binding, subdomain 2		

${}^{a}$ pgm cluster genes were translated using ExPASy, the domains were predicted using InterPRO and the amino acid identities were obtained using BLASTP Web Servers [34,35,36,39,40]; ${}^{b}$ the nucleotide sequence was obtained from GenBank database (strain NIH2624) and modified with GENSCAN [33]; ${}^{c}$ Additional InterPRO domains of PgmA: IPR014030, IPR016039, IPR014031, IPR016035, IPR001227, IPR014043, IPR016036, IPR020807, IPR016040, IPR018201; ${}^{d}$ shared predicted domain; ^e^ not in the vicinity of the cluster or core biosynthesis gene; ${}^{f}$ Accession: CCT64761.1; ${}^{h}$ Accession: CCT64758.1; ${}^{i}$ Accession: CCT64762.1; ${}^{j}$ Accession: CCT65046.1; ${}^{k}$ Accession: CCT64759.1; ${}^{g}$ Accession: CCT64760.1, PgmC and Fsr3 share the same GeneOntology prediction: oxidation reduction process (GO:0055114) and a monooxygenase motif; NA, no significant BLASTP match; COMT: caffeic acid O-methyltransferase; ACP: Acyl carrier protein; SAM: S-adenosyl-L-methionine; MFS: major facilitator superfamily; FAD: flavin adenine dinucleotide; FMN: flavin mononucleotide; CO: carbon monoxide.

## Supplementary Materials

The following are available online at http://www.mdpi.com/2076-2607/5/2/22/s1. Figure S1: Sashimi plots of *pgmB*, *pgmC*, *pgmR* and *pgmD* genes of the *pgm* PKS cluster, Figure S2: Sashimi plots of *pgmE*, *pgmF*, *pgmG* and *pgmH* genes of the pgm PKS cluster, Figure S3: Sashimi plots of ATEG_06202 and ATEG_06212, Figure S4: Sashimi plots of the NRPS-like melanin biosynthase *melA* and tyrosinase *tyrP*, Figure S5: Detailed version of the phylogram in the Figure 4.

## Abbreviations

The following abbreviations are used in this manuscript:

DHN	1,8-dihydroxynaphthalene
DOPA	3,4-dihydroxyphenylalanine
FPKM	fragments per kilobase of exon per million reads mapped
ORF	open reading frame
p.i.	post inoculation
FC	fold change
PKS	polyketide synthase
SAT	starter unit: ACP transacylase
KS	Beta-ketoacyl synthase
AT	Acyl transferase
PT	Polyketide product template
ACP	Acyl carrier
R	Thioester reductase
