# Supplementary material for: Melanisation of Aspergillus terreus—Is Butyrolactone I Involved in the Regulation of Both DOPA and DHN Types of Pigments in Submerged Culture?
Source: Microorganisms. 2017 May 4;5(2):22. doi: 10.3390/microorganisms5020022 (PMC5488093; doi:10.3390/microorganisms5020022)
Supplement: Supplementary file 1 [file microorganisms-05-00022-s001.zip › Figure_S2.pdf]

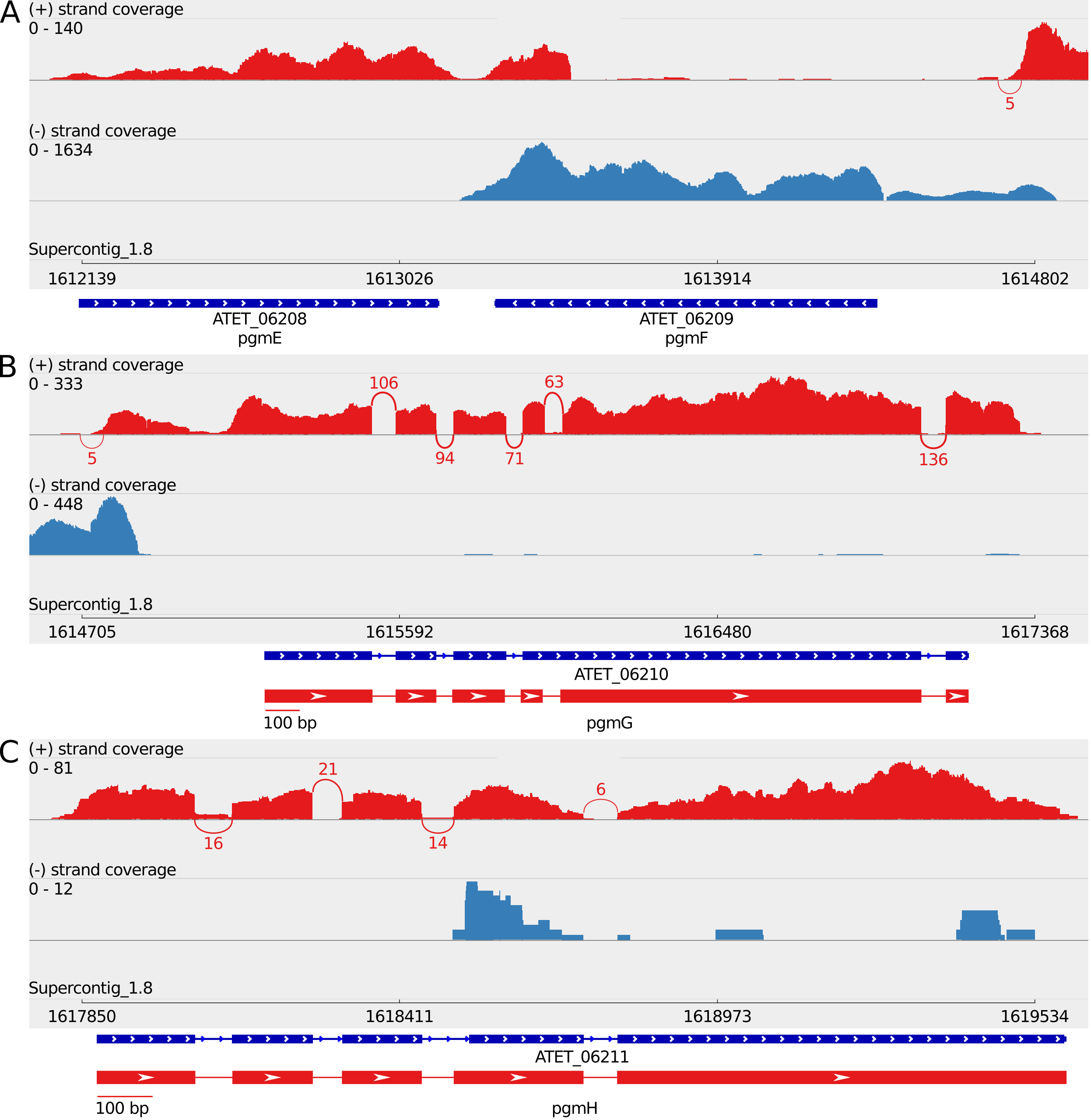

Supplementary figure S2. Transcript read coverages of the pgm gene cluster as visualised by sashimi plots. (**A**) The putative SAM-dependent methyltransferase *pgmE* and the putative quinone reductase *pgmF*, (**B**) the putative MFS family permease *pgmG* and (**C**) the putative FAD/FMN-binding CO dehydrogenase *pgmH*. Plots describe the strand-specific alignment coverage of the read sequences of *A. terreus* MUCL38669 over the corresponding genomic regions of NIH2624 Supercontig_1.8. The curved splice junctions represent the number of spliced reads indicating an intron at that specific location. The RNA to be sequenced was extracted from the set of samples where butyrolactone I had been added at 120 hours post inoculation. The samples were collected at 24, 48, 96, 120, 144 and 216 hours post inoculation and the extracted RNA was pooled prior to sequencing.
