# Supplementary material for: Melanisation of Aspergillus terreus—Is Butyrolactone I Involved in the Regulation of Both DOPA and DHN Types of Pigments in Submerged Culture?
Source: Microorganisms. 2017 May 4;5(2):22. doi: 10.3390/microorganisms5020022 (PMC5488093; doi:10.3390/microorganisms5020022)
Supplement: Supplementary file 1 [file microorganisms-05-00022-s001.zip › Figure_S3.pdf]

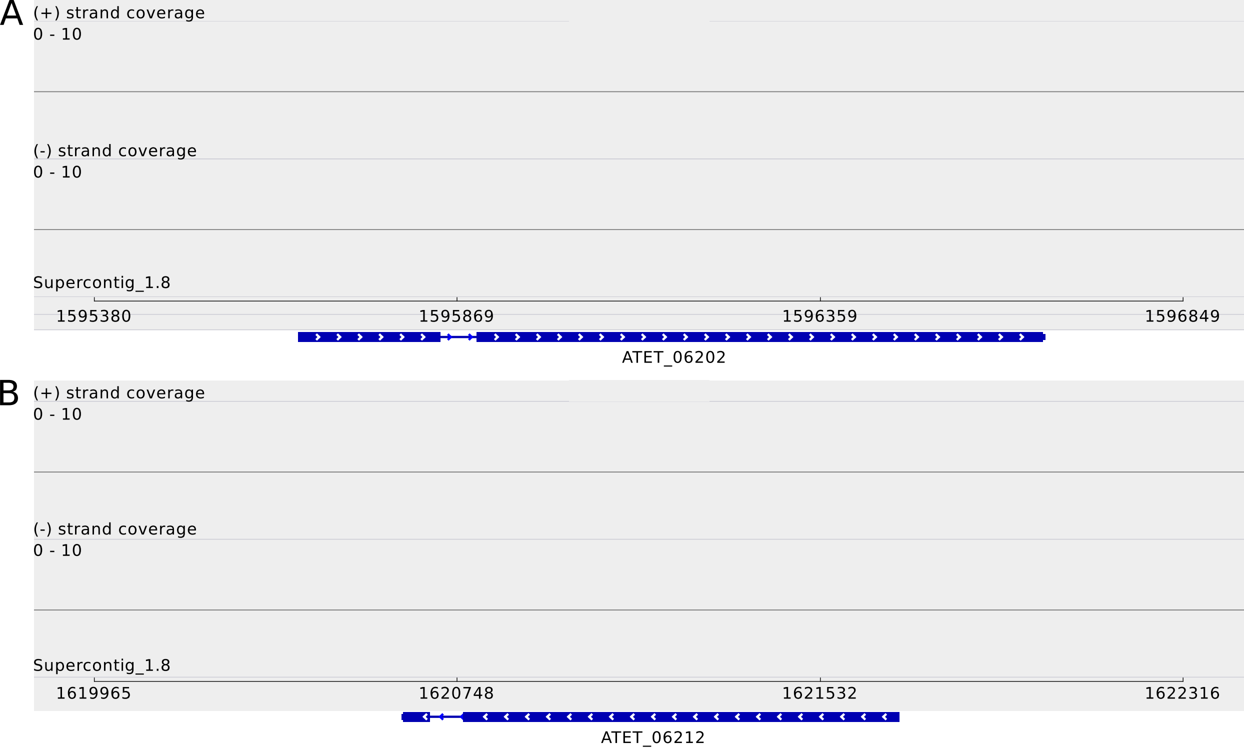

Supplementary figure 3. Sashimi plots of (**A**) ATEG_06202 and (**B**) ATEG_06212 located upstream and downstream of the pgm gene cluster, describing the strand-specific alignment coverage of the read sequences of *A. terreus* strain MUCL38669 over the corresponding genomic regions of NIH2624 Supercontig_1.8. The RNA to be sequenced was extracted from the set of samples where butyrolactone I had been added at 120 hours post inoculation. The samples were collected at 24, 48, 96, 120, 144 and 216 hours post inoculation and the extracted RNA was pooled prior to sequencing.
