# Supplementary material for: Melanisation of Aspergillus terreus—Is Butyrolactone I Involved in the Regulation of Both DOPA and DHN Types of Pigments in Submerged Culture?
Source: Microorganisms. 2017 May 4;5(2):22. doi: 10.3390/microorganisms5020022 (PMC5488093; doi:10.3390/microorganisms5020022)
Supplement: Supplementary file 1 [file microorganisms-05-00022-s001.zip › Figure_S5.pdf]

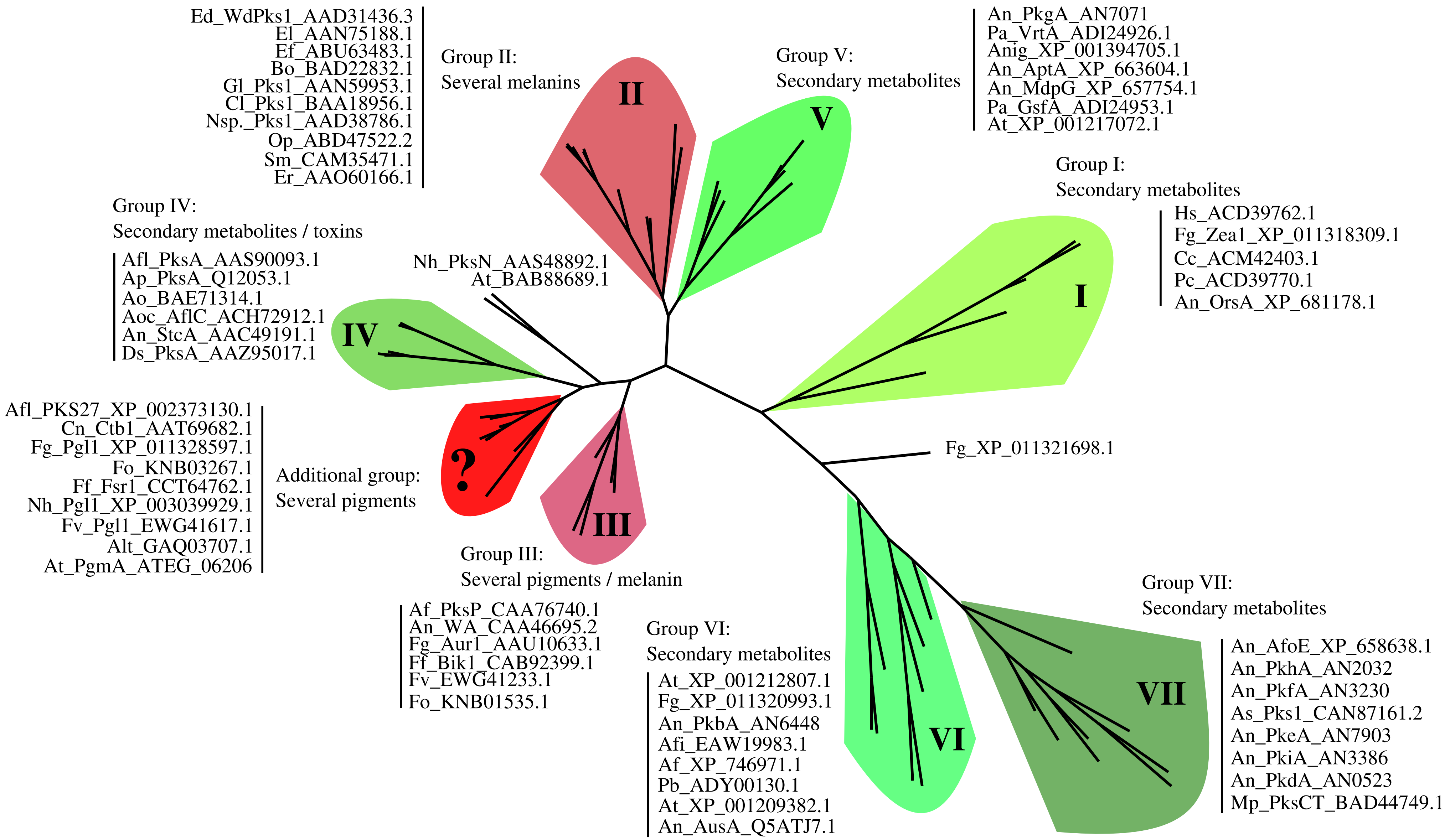

Supplementary figure S5. Figure 4 in details. A phylogenetic schema of various NR-PKS synthases amongst *Ascomycota* species, dividing into eight groups based on the similarity of the KS domain sequences. The groups I-VII are based on the groups presented in the study of Ahuja et al. (2012), and the range of NR-PKS enzymes chosen for this analysis is based on studies of Studt et al. (2012) and Ahuja et al. (2012) [1,2]. The extracted KS domains of the polyketide synthases were compared and aligned to obtain the phylogram as described in the Methods. No outgroup was used. The organisms used in the phylogram: Af; *Aspergillus fumigatus*, Afl; *A.flavus*, Afi; *A. fischeri*, Alt; *A. lentulus*, An; *A. nidulans*, Anig; *A. niger*, Aoc; *A. ochraceoroseus*, Ao; *A. oryzae*, Ap; *A. parasiticus*, At; *A. terreus*; As; *Acremonium strictum*, Bo; *Bipolaris oryzae*, Cc; *Chaetomium chiversii*, Cl; *Colletotrichum lagenarium*, Cn; *Cercospora nicotianae*, Ds; *Dothistroma septosporum*, Ed; *Exophiala dermatitidis*, Ef; *Elsinoe fawcettii*, El; *Exophiala lecanii-corni*, Er; *Endoconidiophora resinifera*, Ff; *Fusarium fujikuroi*, Fg; *F. graminearum*, Fo; *F. oxysporum*, Fs; *F. solani*, Fv; *F. verticillioides*, Gl; *Glarea lozoyensis*, Hs; *Hypomyces subiculosus*, Mp; *Monascus purpureus*, Nh; *Nectria haematococca*, Nsp.; *Nodulisporium* spp. strain ATCC74245, Op; *Ophiostoma piceae*, Pa; *Penicillium aethiopicum*, Pb; *P. brevicompactum*, Pc; *Pochonia chlamydosporia*, Sm; *Sordaria macrospora*. The protein sequences were obtained from the following databases: NCBI RefSeq, GenBank, DDBJ, EMBL and UniProtKB.

[1] Studt, L.; Wiemann, P.; Kleigrewe, K.; Humpf, H.U.; Tudzynski, B. Biosynthesis of Fusarubins Accounts for Pigmentation of Fusarium fujikuroi Perithecia. Appl Environ Microbiol 2012, 78, 4468–4480.
[2] Ahuja, M.; Chiang, Y.M.; Chang, S.L.; Praseuth, M.B.; Entwistle, R.; Sanchez, J.F.; Lo, H.C.; Yeh, H.H.; Oakley, B.R.; Wang, C.C.C. Illuminating the Diversity of Aromatic Polyketide Synthases in Aspergillusnidulans. Journal of the American Chemical Society 2012, 134, 8212–8221.
